# Association Between Lipid Accumulation Product and Cognitive Function in Hypertensive Patients With Normal Weight: Insight From the China H-type Hypertension Registry Study

**DOI:** 10.3389/fneur.2021.732757

**Published:** 2022-02-03

**Authors:** Yanyou Xie, Junpei Li, Guotao Yu, Xinlei Zhou, Wei Zhou, Lingjuan Zhu, Tao Wang, Xiao Huang, Huihui Bao, Xiaoshu Cheng

**Affiliations:** ^1^Department of Cardiovascular, The Second Affiliated Hospital of Nanchang University, Nanchang, China; ^2^Urban Medical Institutions, Jiangwan Public Health Center, Wuyuan, China; ^3^Center for Cardiovascular Disease Prevention and Treatment, The Second Affiliated Hospital of Nanchang University, Nanchang, China

**Keywords:** lipid accumulation product, cognitive function, mini-mental state examination, hypertension, normal-weight

## Abstract

**Background:**

Hypertension is a major cardiovascular risk factor for cognitive impairment. Lipid accumulation product (LAP), an index that represents fat overaccumulation in the body, has been shown to be associated with cardiovascular disease. Nevertheless, the relationship between LAP and cognitive function in hypertensive patients with normal weight has been infrequently studied.

**Objective:**

This study aimed to assess the relationship between LAP and cognitive function in hypertensive patients with normal weight.

**Methods:**

This study included 5,542 Chinese hypertensive patients with normal weight. Cognitive function was evaluated using the Mini-Mental State Examination (MMSE). The relationship between LAP and MMSE scores was evaluated using multiple linear regression.

**Results:**

The mean age of the participants was 64.8 ± 9.3 years, and 2,700 were men (48.7%). The mean MMSE score was 24.5 ± 5.1 in men and 19.2 ± 6.5 in women. The mean LAP was 26.2 ± 25.5 in men and 42.5 ± 34 in women. Log_10_-LAP showed a significant positive association with MMSE score (men: β = 0.69, 95% CI 0.14–1.24, *p* = 0.015; women: β = 1.03, 95% CI 0.16–1.90, *p* = 0.020). When LAP was divided into 3 groups according to tertiles, participants in the third LAP tertile had higher MMSE scores for both men (*p* for trend = 0.04) and women (*p* for trend = 0.015).

**Conclusion:**

LAP showed an independent positive association with MMSE in Chinese hypertensive patients with normal weight.

## Introduction

According to the World Alzheimer Report 2015, ~46.8 million people are living with dementia worldwide, and this number is predicted to exceed 131.5 million by 2050 (1). A large-sample, multiregional study was conducted in 2019, and it showed that the prevalence of dementia was 5.60% (3.50–7.60) for individuals aged ≥65 years in China (2). Dementia is a notable burden for families and society, and substantial evidence indicates that hypertension is a crucial risk factor for dementia. Several cohort studies have suggested that high blood pressure in middle-aged individuals is linked to an increased risk of cognitive decline over time (3–5).

Obesity is considered a risk factor for dementia (6, 7). However, the association between obesity and the risk of dementia is unclear. In late life, there are less apparent adverse effects of obesity, and potential protective effects are observed. A recent meta-analysis reported a positive association between obesity in middle age and subsequent dementia, but a negative association was noted between these two parameters in old age (8). The limitations of traditional anthropometric measurements used to evaluate obesity are obvious. Obesity is usually distinguished by the excessive accumulation of fatty tissue. Body mass index (BMI) cannot be used to differentiate between adipose and lean tissue, and although waist circumference (WC) is a fair indicator of abdominal adipose tissue, it is still unable to correctly reflect whole-body adipose tissue (9, 10). Although imaging examinations are the “gold standard” for the assessment of adipose tissue, they are too costly for extensive use. Lipid accumulation product (LAP) is an index, that is calculated from WC and triglyceride (TG) levels, and it has been proposed as a simple and economical method to evaluate lipid accumulation (11). Studies have indicated that LAP is highly correlated with metabolic syndromes (12), type 2 diabetes mellitus (13), stroke (14), and arterial stiffness (15). Thus, researchers have paid increasing attention to the clinical significance of LAP.

Obesity often occurs along with metabolic abnormality, however, some persons with normal weight may have substantial metabolic disorders that are similar to that of obese people. These individuals are known as metabolically obese normal-weight (MONW). Epidemiologic studies have reported that MONW individuals account for ~20% of the normal-BMI population (16, 17). A recent study demonstrated that LAP is an effective marker for identifying the MONW phenotype in Chinese adults (17). Obesity is often associated with hypertension, as either a causative factor or a concomitant disease. Elevated blood pressure has a direct effect on cognitive performance, several relevant studies have reported that hypertension in midlife increased the risk of cognitive dysfunction over time (3–5). However, it remains unknown whether metabolic abnormality in normal-weight individuals may have an impact on cognition function in the hypertensive population. Consequently, this study sought to assess the relationship between LAP and cognitive function in hypertensive participants with a normal weight.

## Methods

### Participants

The data of this study were derived from the China H-type Hypertension Registry Study (registration number: ChiCTR1800017274). Briefly, the study was a large-scale, real-world observational registry study that aimed to create a national registry of patients with H-type hypertension, investigate the prevalence and control of H-type hypertension in China, and explore related factors that would affect disease prognosis. Details of the methodology of this study had been published elsewhere (18). The enrolled participants fulfilled the following criteria: (1) age ≥ 18 years and (2) hypertension defined as systolic blood pressure (SBP) ≥ 140 mm Hg and/or diastolic blood pressure (DBP) ≥ 90 mm Hg, self-reported diagnosis of hypertension previously, or receiving antihypertensive medication at baseline. Participants were excluded based on the following conditions: (1) inability to provide informed consent because of psychological or nervous system impairment and (2) follow-up was notably difficult to complete according to the study protocol. The study was approved by the Ethics Committee of the Biomedical Institute of Anhui Medical University. Written informed consent was obtained from all the study participants.

**Figure 1:**
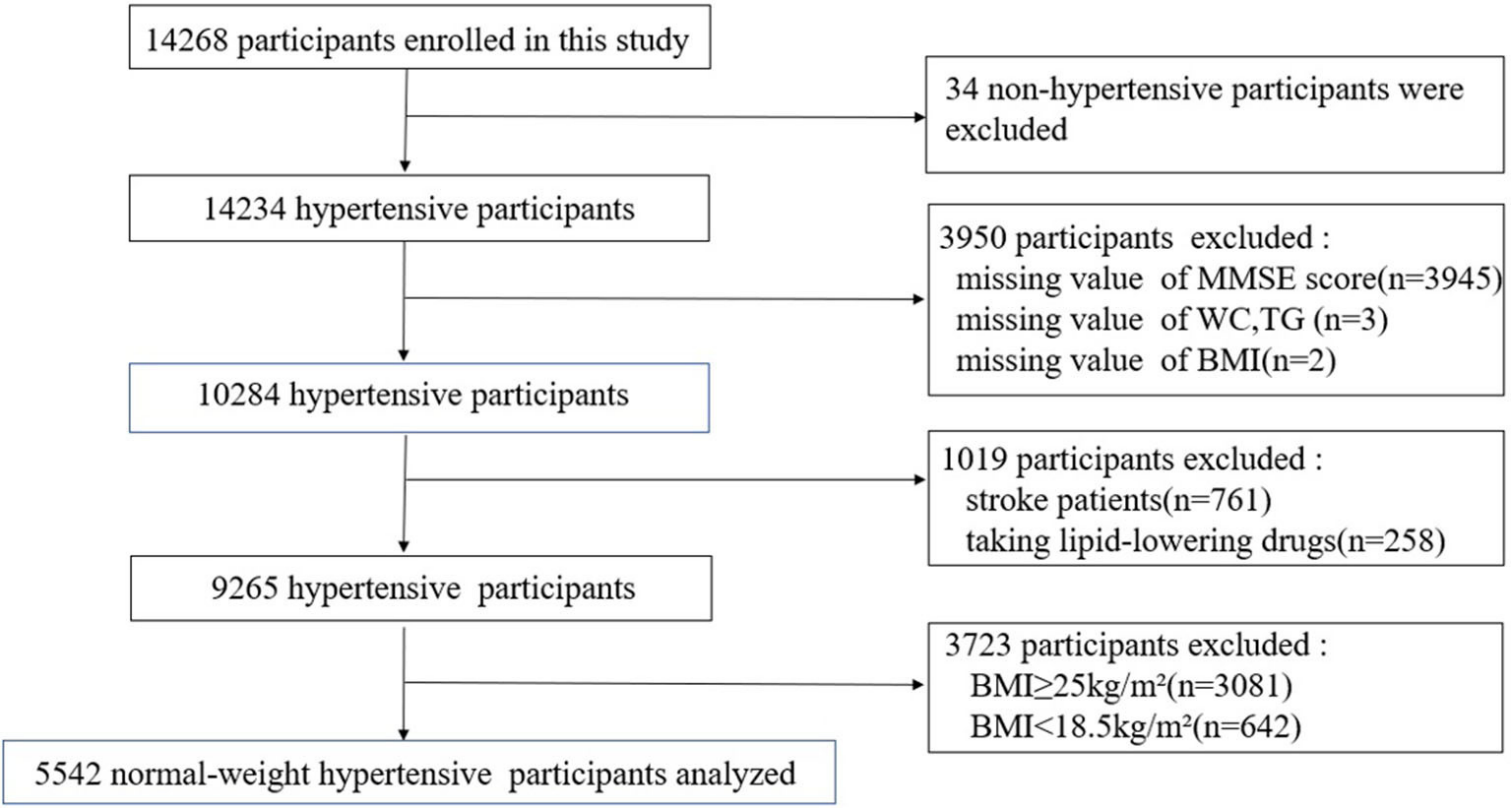
Flow chart of the study participants.

A total of 10,255 hypertensive patients completed the Mini-Mental State Examination (MMSE) questionnaire. We excluded participants with missing LAP (*n* = 3) and BMI (*n* = 2) data, and those with stroke (*n* = 761), lipid-lowering drugs (*n* = 258), BMI ≥ 25 kg/m^2^ (*n* = 3,081), or BMI < 18.5 kg/m^2^ (*n* = 624). Ultimately, 5,542 subjects were included in the analysis (Figure 1).

### Data Collection

The research staff used questionnaires to gather information, namely, demographic characteristics, lifestyle data, medication history, medical history, and MMSE. The anthropometric indices, namely, height, weight, WC, SBP, and DBP, were measured by trained investigators. Blood pressure was obtained by electronic sphygmomanometers on the right arm placed at the heart level in the sitting position after the participants rested for at least 5 min; the blood pressure was calculated as the mean of three consecutive measurements.

After an overnight fast of at least 8 h, venous blood samples were collected from all study participants by trained research staff and sent to the Biaojia Biotechnology Laboratory, which is located in Shenzhen, China. Lipid profiles [total cholesterol, TG, low-density lipoprotein-cholesterol (LDL-C), and high-density lipoprotein-cholesterol (HDL-C)], fasting blood glucose, homocysteine, and creatinine levels were measured using an automatic clinical assay.

Lipid accumulation product (LAP) was calculated based on fasting TG and WC values using the following formula: LAP = [WC (cm) – 65] × TG (mmol/l) for men and [WC (cm) – 58] × TG (mmol/l) for women (11). A total of 104 men had WC ≤ 65 cm and 16 women had WC ≤ 58 cm. WC values for men that were ≤ 65 cm were reassigned to 66 cm and those WC values for women that were ≤ 58 cm were reassigned to 59 cm to generate a LAP-value >0 and enable the logarithmic transformation of LAP. Diabetes was defined as either fasting blood glucose ≥7.0 mmol/l, self-report of a physician diagnosis, or self-report use of antidiabetic medication.

### Assessment of Cognitive Function

Cognitive function was assessed by the Chinese MMSE version in this study. This tool has been widely used because of its proven high reliability and has been validated for use in the Chinese population (19). MMSE consisted of the following cognitive domains: orientation (10 points), immediate recall (3 points), short-term verbal memory (3 points), visuospatial memory (1 point), language (8 points), and attention and calculation (5 points). MMSE scores ranged from 0 to 30 points, with higher scores indicating better cognition.

### Covariate

The selected covariates included age, sex, education (primary school graduate or below, middle/high/special school, and college graduate or above), current drinking (yes or no), current smoking (yes or no), SBP, DBP, diabetes (yes or no), coronary heart disease (yes or no), antihypertensive drug (yes or no), homocysteine, total cholesterol, HDL-C, LDL-C, and estimated glomerular filtration rate (eGFR).

### Statistical Analysis

The baseline characteristics of the total population are presented as mean ± SD for continuous variables and as percentages (%) for categorical variables. ANOVA or the chi-squared test was used to examine the differences between the groups by LAP tertiles. Because LAP exhibited a skewed distribution, logarithmic data were used for the statistical analyses. Because LAP and the MMSE score have significant discrepancies with respect to sex, all subsequent analyses were performed separately for men and women. Multivariate linear regression models were used to evaluate the association of LAP with the MMSE score, and the results are presented as β and 95% CIs. All the analyses consisted of three statistical models. The crude model was not adjusted, model 1 was adjusted for age and education, and model 2 was adjusted for age, education, drinking status, smoking status, SBP, DBP, CHD, diabetes, an antihypertensive drug, homocysteine, total cholesterol, LDL-C, HDL-C, and eGFR. Trend tests were conducted using LAP tertile categories as continuous data. The dose–response association of LAP and the MMSE score was performed using a fitted smoothing curve. Stratified analyses were performed in various subgroups, namely, age, drinking status, smoking status, SBP, DBP, diabetes, total cholesterol, and eGFR.

R version 3.4.3 (R Foundation for Statistical Computing, Vienna, Austria) and EmpowerStates (www.empowerstats.com) were used to conduct the data analyses. Only *p* < 0.05 (two-sided) were deemed as statistically significant.

## Results

### Baseline Characteristics

Of the 5,542 participants, 2,700 (48.7%) were men and 2,842 (51.3%) were women, and the mean age of the participants was 64.8 ± 9.3 years. The baseline characteristics of the study participants were distributed according to sex and LAP tertiles as presented in Table 1. The LAP tertile ranges were ≤ 13.3, 13.3–27.2, and ≥27.2 in the men group and ≤ 25.1, 25.1–45.2, and ≥45.2 in the women group. In the men group, participants with higher LAP levels had higher BMI, WC, DBP, eGFR, TG, total cholesterol, and MMSE scores, and were more likely to have diabetes and a higher educational level. However, they had lower values for age, SBP, and HDL-C and were less likely to smoke. In the women group, participants with higher LAP levels had higher BMI, WC, DBP, TG, total cholesterol, and MMSE scores, and were more likely to have diabetes. In contrast, participants with higher LAP levels had lower values of age and HDL-C.

**Table 1:** Baseline characteristics of study participants.

	**Male**				**Female**			
	**LAP index tertiles**				**LAP index tertiles**			
Characteristics	<13.3	13.3–27.2	≥27.2	*P*-value	<25.1	25.1–45.2	≥45.2	*P-*value
Number	900	900	900		947	947	948	
Age, years	67.3 ± 8.8	65.9 ± 9.4	62.3 ± 9.5	<0.001	65.2 ± 9.9	64.7 ± 9.3	63.6 ± 8.4	<0.001
BMI, kg/m^2^	20.6 ± 1.4	22.2 ± 1.5	23.3 ± 1.3	<0.001	21.2 ± 1.6	22.4 ± 1.6	23.0 ± 1.4	<0.001
WC, cm	74.5 ± 4.6	81.9 ± 4.4	87.0 ± 4.5	<0.001	74.4 ± 5.4	80.8 ± 5.2	84.4 ± 6.1	<0.001
SBP, mmHg	146.7 ± 19.0	146.2 ± 17.5	144.3 ± 16.4	0.012	149.5 ± 17.8	149.6 ± 16.7	150.3 ± 17.4	0.553
DBP, mmHg	87.7 ± 10.6	89.1 ± 10.5	91.2 ± 10.3	<0.001	87.0 ± 10.7	87.3 ± 9.9	88.4 ± 10.1	0.005
Current smoking, *n* (%)	486 (54.0)	457 (50.8)	430 (47.8)	0.031	64 (6.8)	56 (5.9)	63 (6.6)	0.718
Current drinking, *n* (%)	394 (43.8)	366 (40.7)	393 (43.7)	0.318	51 (5.4)	56 (5.9)	44 (4.6)	0.463
Education status, *n* (%)				<0.001				0.471
Primary school graduate or below	694 (77.1)	629 (69.9)	540 (60.0)		868 (91.7)	878 (92.7)	887 (93.6)	
Middle/high/special school	195 (21.7)	245 (27.2)	321 (35.7)		74 (7.8)	67 (7.1)	58 (6.1)	
College graduate or above	11 (1.2)	26 (2.9)	39 (4.3)		5 (0.5)	2 (0.2)	3 (0.3)	
Living conditions, *n* (%)				0.001				0.007
Low	102 (11.3)	115 (12.8)	142 (15.8)		127 (13.4)	107 (11.3)	118 (12.4)	
Moderate	599 (66.6)	633 (70.3)	613 (68.1)		596 (62.9)	670 (70.7)	643 (67.8)	
High	199 (22.1)	152 (16.9)	145 (16.1)		224 (23.7)	170 (18.0)	187 (19.7)	
Marital status, *n* (%)				<0.001				<0.001
Never married	15 (1.7)	21 (2.3)	14 (1.6)		1 (0.1)	0 (0.0)	1 (0.1)	
Married	743 (82.6)	758 (84.2)	802 (89.1)		642 (67.8)	710 (75.0)	751 (79.2)	
Other	142 (15.8)	121 (13.4)	84 (9.3)		304 (32.1)	237 (25.0)	196 (20.7)	
Physical activity, *n* (%)				0.001				0.709
Mild	431 (47.9)	476 (52.9)	506 (56.2)		518 (54.7)	528 (55.8)	544 (57.4)	
Moderate	235 (26.1)	232 (25.8)	227 (25.2)		224 (23.7)	223 (23.5)	203 (21.4)	
Vigorous	234 (26.0)	192 (21.3)	167 (18.6)		205 (21.6)	196 (20.7)	201 (21.1)	
CHD, *n* (%)	42 (4.7)	47 (5.2)	52 (5.8)	0.571	49 (5.2)	53 (5.6)	43 (4.5)	0.572
Diabetes, *n* (%)	63 (7.0)	107 (11.9)	173 (19.2)	<0.001	95 (10.0)	158 (16.7)	264 (27.8)	<0.001
eGFR, mL/min per 1.73 m^2^	82.6 ± 20.2	83.9 ± 19.2	85.6 ± 19.8	0.006	87.9 ± 18.6	87.6 ± 18.6	87.1 ± 19.8	0.64
Laboratory results								
Triglyceride, mmol/L	0.9 ± 0.3	1.2 ± 0.4	2.4 ± 1.3	<0.001	1.1 ± 0.3	1.6 ± 0.4	3.0 ± 1.5	<0.001
Total cholesterol, mmol/L	4.7 ± 0.9	4.9 ± 1.0	5.2 ± 1.2	<0.001	5.1 ± 1.0	5.3 ± 1.1	5.5 ± 1.2	<0.001
HDL-C, mmol/L	1.7 ± 0.4	1.5 ± 0.4	1.4 ± 0.3	<0.001	1.7 ± 0.4	1.5 ± 0.4	1.4 ± 0.3	<0.001
Homocysteine, mmol/L	20.3 ± 12.7	21.1 ± 14.4	19.7 ± 13.0	0.077	15.7 ± 7.8	15.6 ± 6.8	15.6 ± 6.9	0.957
MMSE score	23.7 ± 5.3	24.3 ± 5.0	25.6 ± 4.8	<0.001	18.6 ± 6.6	19.3 ± 6.4	19.5 ± 6.4	0.006

*LAP, lipid accumulation product; BMI, body mass index; SBP, systolic blood pressure; DBP, diastolic blood pressure; CHD, coronary heart disease; HDL, high-density lipoprotein cholesterol*.

### Association Between LAP and MMSE Score

**Figure 2:**
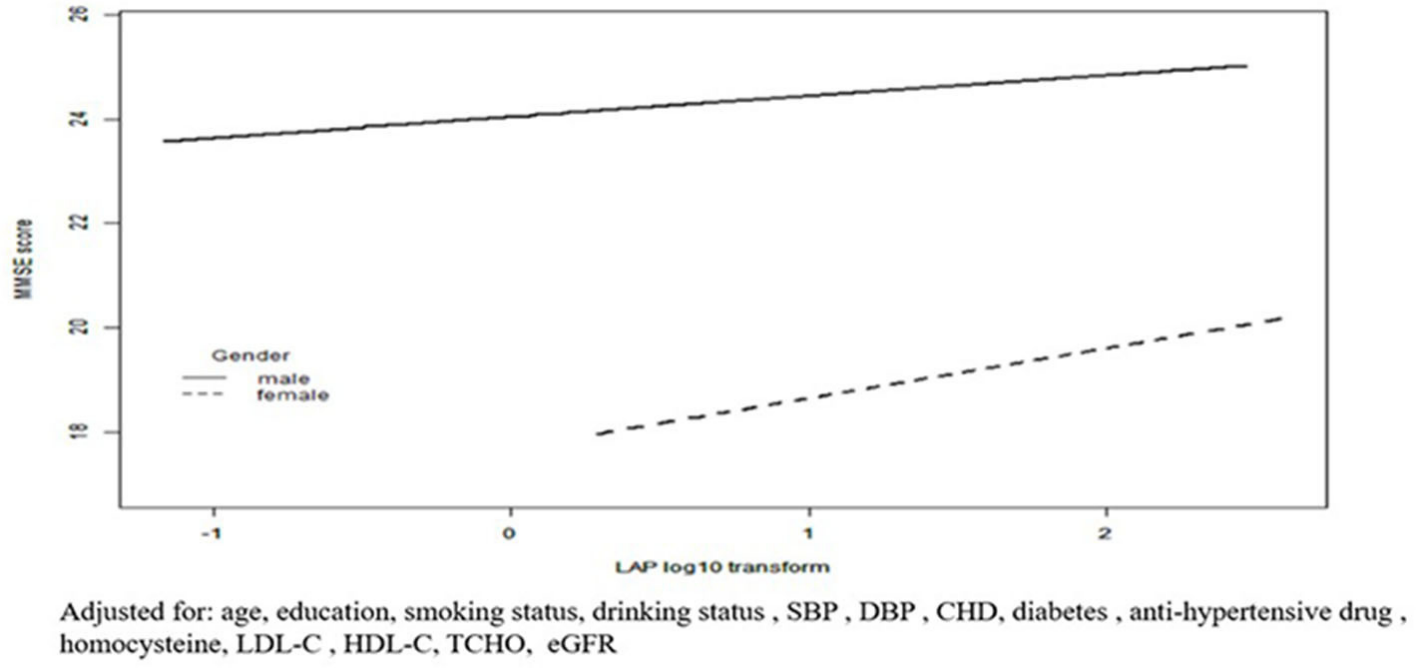
Fitted curves for LAP index as a common logarithm-transformed continuous variable and cognitive function represented by MMSE scores. Adjusted for: age, education, smoking status, drinking status, SBP, DBP, CHD, diabetes, anti-hypertensive drug, homocysteine, LDL-C, HDL-C, TCHO, eGFR.

The results of the linear regression analysis investigating the association of LAP with MMSE score are shown in Table 2. In the men group, log_10_-LAP (β = 2.03, 95% CI 1.55–2.51) had a significant positive association with the MMSE score in the crude model. After adjustment for age and education, this association remained significant (β = 1.03, 95% CI 0.57–1.48). With additional adjustment for smoking status, drinking status, SBP, DBP, CHD, diabetes, an antihypertensive drug, homocysteine, total cholesterol, HDL-C, LDL-C, and eGFR, this association was still significant (β = 0.69, 95% CI 0.14–1.24). Likewise, The regression coefficient (β) and 95% CI of the relationship of log_10_-LAP with MMSE score was (1.23, 0.45–2.02), (1.11, 0.39–1.82), and (1.03, 0.16–1.90) for the crude model, model 1, and model 2, respectively, in the women group. When LAP was assessed as tertiles (Table 2), LAP was positively associated with MMSE score among the men (*p* for trend = 0.04) and women (*p* for trend = 0.015). Figure 2 shows a positive association between LAP and MMSE score in both men and women.

**Table 2:** Association between LAP as a common logarithm-transformed continuous variable and cognitive function represented by MMSE scores.

**Lg LAP index**	***N***	**MMSE scores (Mean ± SD)**	**Crude model**		**Model 1**		**Model 2**	
			**β (95%CI)**	***P*-value**	**β (95%CI)**	***P*-value**	**β (95%CI)**	***P*-value**
Male								
Continuous	2,700	24.5 ± 5.1	2.03 (1.55, 2.51)	<0.001	1.03 (0.57, 1.48)	<0.001	0.69 (0.14, 1.24)	0.015
Tertiles								
T1	900	23.7 ± 5.3	0(ref.)		0(ref.)		0(ref.)	
T2	900	24.3 ± 5.0	0.62 (0.16, 1.09)	0.009	0.26 (–0.17, 0.69)	0.233	0.08 (–0.37, 0.52)	0.732
T3	900	25.6 ± 4.8	1.88 (1.41, 2.34)	<0.001	0.95 (0.51, 1.39)	<0.001	0.57 (0.06, 1.09)	0.030
P for trend			<0.001		<0.001		0.040	
Female								
Continuous	2,842	17.6 ± 6.4	1.23 (0.45, 2.02)	0.002	1.11 (0.39, 1.82)	0.002	1.03 (0.16, 1.90)	0.020
Tertiles								
T1	947	18.6 ± 6.6	0(ref.)		0(ref.)		0(ref.)	
T2	947	19.3 ± 6.4	0.71 (0.13, 1.29)	0.017	0.71 (0.18, 1.24)	0.009	0.66 (0.11, 1.21)	0.020
T3	948	19.5 ± 6.4	0.90 (0.32, 1.48)	0.003	0.84 (0.31, 1.37)	0.002	0.77 (0.15, 1.38)	0.015
*P* for trend			<0.001		<0.001		0.015	

*Model 1, adjusted for age, education*.

*Model 2, adjusted for age, education, smoking status, drinking status, SBP, DBP, CHD, diabetes, anti-hypertensive drug, homocysteine, LDL-C, HDL-C, TCHO, eGFR*.

### Subgroup Analyses

In the stratified analysis, we further assessed the relationship between LAP and MMSE scores according to various subgroups. As presented in Tables 3, 4, except for the age subgroup in women (*p* for interaction = 0.008), significant differences were not found for drinking status, smoking status, diabetes, SBP, DBP, total cholesterol, and eGFR regardless of sex (all *P* for interaction > 0.05). Women ≥65 years showed significantly higher MMSE scores (β = 0.98, 95% CI 0.04–1.92) compared to women <65 years.

**Table 3:** Stratified analyses of association between LAP as a common logarithm-transformed continuous variable and cognitive function represented by MMSE scores in male.

**Subgroups**	***N***	**MMSE Score (Mean ± SD)**	**β 95%(CI)**		***P*-interaction**
Age, year				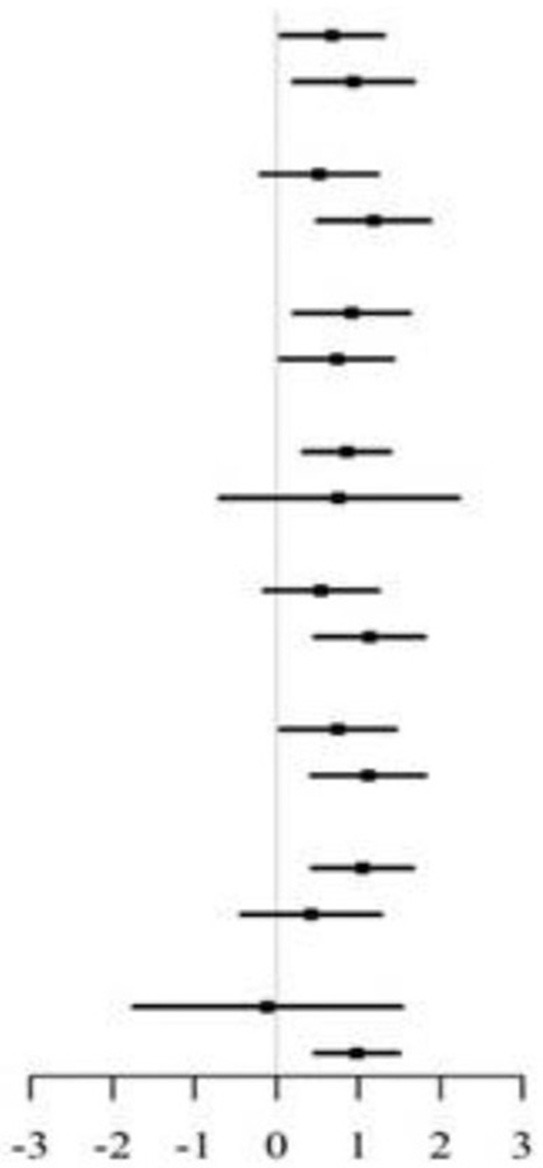	0.543
>65	1183	25.8 ± 4.3	0.68 (0.06, 1.30)		
≤ 65	1517	23.6 ± 5.5	0.94 (0.21, 1.67)		
Current smoking					0.11
No	1327	24.7 ± 5.0	0.52 (−0.19, 1.23)		
Yes	1373	24.3 ± 5.2	1.19 (0.50, 1.87)		
Current drinking					0.618
No	1547	24.3 ± 5.2	0.92 (0.22, 1.62)		
Yes	1153	24.8 ± 4.9	0.74 (0.05, 1.42)		
Diabetes					0.567
No	2357	24.5 ± 5.1	0.86 (0.33, 1.38)		
Yes	343	25.1 ± 5.0	0.76 (−0.69, 2.22)		
SBP, mmHg					0.425
<140	1030	25.2 ± 4.7	0.54 (−0.15, 1.24)		
≥140	1670	24.1 ± 5.3	1.14 (0.47, 1.80)		
DB, mmHg					0.908
<90	1330	24.3 ± 5.1	0.75 (0.05, 1.45)		
≥90	1370	24.8 ± 5.1	1.12 (0.43, 1.81)		
TCHO, mmol/L					0.168
<5.2	1726	24.4 ± 5.1	1.05 (0.44, 1.66)		
≥5.2	974	24.8 ± 5.1	0.42 (−0.43, 1.27)		
eGFR, mL/min per 1.73 m^2^					0.287
<60	347	23.1 ± 6.0	−0.11(−1.74,1.52)		
≥60	2353	24.7 ± 4.9	0.98 (0.47, 1.49)		

*Adjusted for: age, education, smoking status, drinking status, SBP, DBP, CHD, diabetes, anti-hypertensive drug, homocysteine, LDL-C, HDL-C, TCHO, eGFR*.

**Table 4:** Stratified analyses of association between LAP as a common logarithm-transformed continuous variable and cognitive function represented by MMSE scores in female.

**Subgroups**	***N***	**MMSE Score (Mean ± SD)**	**β 95%(CI)**		***P*-interaction**
Age, year				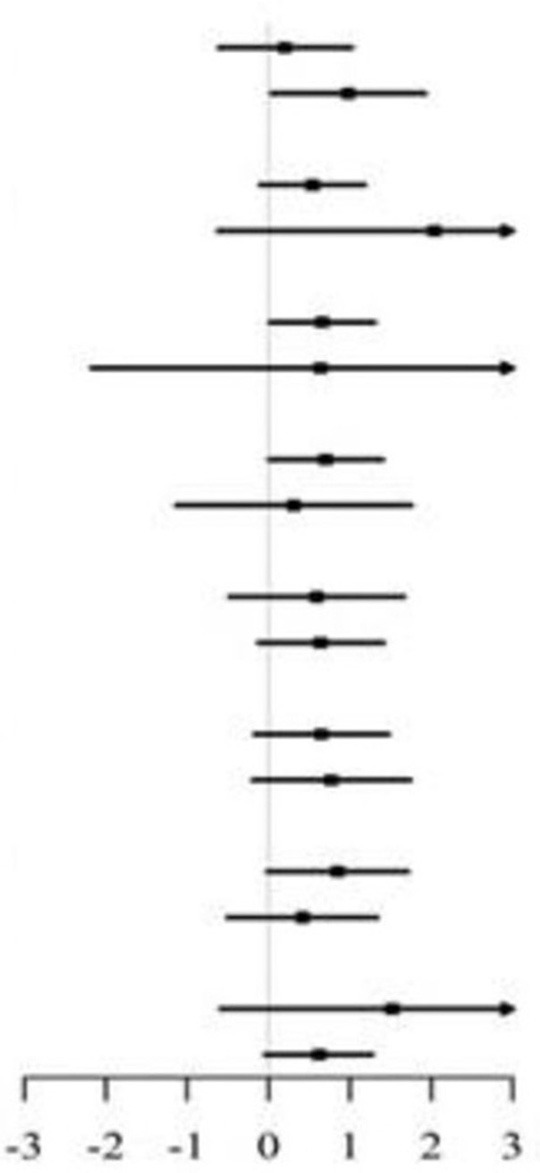	0.008
<65	1350	20.9 ± 6.1	0.20 (−0.61, 1.02)		
≥65	1492	17.6 ± 6.4	0.98 (0.04, 1.92)		
Current smoking					0.065
No	2659	19.2 ± 6.5	0.54 (−0.10, 1.18)		
Yes	183	18.1 ± 6.3	2.04 (−0.62, 4.70)		
Current drinking					0.782
No	2691	19.2 ± 6.5	0.66 (0.02, 1.30)		
Yes	151	18.0 ± 6.4	0.64 (−2.17, 3.46)		
Diabetes					0.487
No	2325	19.2 ± 6.5	0.70 (0.01, 1.40)		
Yes	517	19.1 ± 6.4	0.31 (−1.13, 1.75)		
SBP, mmHg					0.576
<140	786	20.6 ± 6.2	0.59 (−0.48, 1.66)		
≥140	2056	18.6 ± 6.5	0.64 (−0.12, 1.41)		
DBP, mmHg					0.579
<90	1642	18.9 ± 6.4	0.65 (−0.17, 1.47)		
≥90	1,200	19.5 ± 6.5	0.77 (−0.19, 1.74)		
TCHO, mmol/L					0.391
<5.2	1386	19.1 ± 6.4	0.85 (−0.01, 1.71)		
≥5.2	1456	19.2 ± 6.5	0.42 (−0.50, 1.33)		
eGFR, mL/min per 1.73 m^2^					0.64
<60	266	17.7 ± 6.8	1.52 (−0.59, 3.62)		
≥60	2576	19.3 ± 6.4	0.62 (−0.04, 1.27)		

*Adjusted for: age, education, smoking status, drinking status, SBP, DBP, CHD, diabetes, anti-hypertensive drug, homocysteine, LDL-C, HDL-C, TCHO, eGFR*.

## Discussion

In this cross-sectional study, we found a significant positive association between LAP and cognitive function in normal-weight hypertensive patients. In addition, our results suggested that the positive relationship between LAP and cognitive function was more prominent in women aged ≥65 years.

Several studies have assessed the impact of obesity on cognitive function. A prospective study conducted by Whitmer et al. showed that obese individuals in midlife showed an increased risk of future cognitive impairment among adults in America (20). However, another observational study performed by Hou et al. demonstrated that being overweight was independently associated with a reduced risk of cognitive impairment in elderly Chinese individuals (21). In addition, a longitudinal study by Luchsinger et al. reported the lack of an association between BMI and late-life dementia in the elderly in America (22). The relationship between adiposity, defined using BMI in the above studies, and cognitive function was analyzed in middle and late life, and the results were conflicting. The discrepancies in these findings might be attributed to variations in countries, races, study sizes, adjusted variables, or participant ages. In addition, the elderly usually have decreased muscle weight and increased fat weight. However, BMI does not reflect body fat accumulation, and it does not differentiate between adipose and lean tissue. LAP represents both the anatomical and physiological changes associated with fat overaccumulation in the body. Compared to BMI, LAP is more appropriate for explaining the relationship between obesity and cognitive function. It is well-known that increased weight might be due to the enlargement of lean tissues or systemic overload of fluid. A longitudinal Icelandic study also showed that a higher amount of muscle was associated with a lower risk of dementia in men and women (23). This may explain why the relationship between BMI and cognitive function was inconsistent in previous studies.

Therefore, it is important to explore the relationship between LAP and cognitive function among normal-weight individuals. However, data on the role of LAP in cognitive function among hypertensive populations are scarce, and our study is the first to explore the association between LAP and cognitive function in hypertensive patients. Our results were in line with a prospective study conducted in 16,791 hypertensive participants in China, which showed that obesity defined by BMI was independently related to slower cognitive decline in Chinese adult patients with hypertension (24). Furthermore, Yu et al. (25) conducted a cross-sectional study to examine the relationship between LAP and mild cognitive impairment in 220 patients with type 2 diabetes and found that higher LAP was related to an increased risk of mild cognitive impairment in patients with type 2 diabetes. The inconsistent results might be due to the differences in the study population, and the sample size was relatively small; thus, large sample size may yield clearer results. While the sample size was 5,542 in this study, our result that higher LAP was related to better cognitive function in hypertensive participants seems plausible.

A possible mechanism underlying the relationship between LAP and cognitive function may be linked to leptin, which is produced by adipose tissue. Some studies have indicated that leptin could promote synaptogenesis and neurogenesis in the hippocampus, which is related to learning and memory, thus facilitating cognitive function (26). Tau hyperphosphorylation and β-amyloid peptide deposition have been implicated in the pathogenesis of Alzheimer's disease (27). Several laboratory studies have indicated that leptin reduces tau phosphorylation and β-amyloid peptide production. These attributes of leptin in the brain confer protection against the risk of Alzheimer's disease (26). Another possible explanation is that the accumulation of low adipose tissue in the body may be attributed to preclinical dementia and other preexisting illnesses (28). This could directly or indirectly disrupt the energy balance through neuronal dysfunction and neurodegeneration. These include disturbances in cognition, motion, psychology, and pathology of the energy balance center in the hypothalamus. People may forget to eat or lack the desire to eat because of the loss of olfaction and gustation.

In our study, older women (aged ≥ 65 years) had larger regression coefficients for LAP in relation to MMSE scores than younger women. The sex difference in estrogen is a possible explanation for this phenomenon. Adipose tissue is the major source of estrogen in postmenopausal women (29, 30). Estrogen has been demonstrated to play an important role in the bioenergetic system of the brain, namely, glucose transport, glycolysis, the tricarboxylic citric acid cycle, oxidative phosphorylation, and ATP production (31). In addition, body fat loss preceding the onset of the clinical syndrome of dementia by as much as 10 years may also be related to this phenomenon (32). Therefore, the higher LAP in older women with normal weight was associated with better cognitive function than in younger women.

This study has several limitations. First, the sensitivity and specificity of the MMSE for identifying mild cognitive impairment may be limited. Our study participants mainly came from the rural areas in China, where people were more likely to be less well-educated. While the MMSE is proved to be a useful tool for assessing cognitive function in the low educated population (33), so it may be more appropriate for the population of this study. Further research combining MMSE and other cognitive function rating scales, such as the Montreal Cognitive Assessment or Mini-Cog, is needed in the future to validate the result. Second, this was a cross-sectional study, and it failed to identify any causal relationship between LAP and cognitive function in normal-weight hypertensive individuals. Third, although we carefully adjusted for several important confounders using various statistical models, there may still be potential confounding factors that were not considered. Fourth, lack of information on the type of glucose-lowering drugs for diabetics. Thus, it was unable to evaluate the impact of specific glucose-lowering drugs in this study. Finally, this study was conducted in hypertensive populations, our findings may not be appropriate to generalize to other populations. However, hypertension is a significant risk factor for dementia, and more attention needs be paid to this population.

## Conclusion

Overall, LAP demonstrated a significant positive relationship with cognitive function in normal-weight hypertensive persons. The results indicate that appropriate lipid accumulation in normal-weight individuals is associated with better cognitive function. The results obtained from this study may be valuable for the prevention of cognitive dysfunction.

## Data Availability Statement

The raw data supporting the conclusions of this article will be made available by the authors, without undue reservation.

## Ethics Statement

The studies involving human participants were reviewed and approved by Ethics Committee of the Biomedical Institute of Anhui Medical University. The patients/participants provided their written informed consent to participate in this study.

## Author Contributions

XC and HB contributed to the design of the study and reviewed the article. YX and JL were responsible for the data analysis and edited this article. XZ contributed to the article and data review. GY contributed to the data collection. LZ and TW contributed to the data interpretation. XH and WZ revised the article for scientific and logical accuracy. All authors read and approved the final version of the manuscript.

## Funding

This study was supported by the National Natural Science Foundation of China (81760049), Jiangxi Science and Technology Innovation Platform Project (20165BCD41005), National Key R&D Program of China (2018YFC1312902), and Key Project of the Education Department of Jiangxi Province (GJJ170013).

## Conflict of Interest

The authors declare that the research was conducted in the absence of any commercial or financial relationships that could be construed as a potential conflict of interest.

## Publisher's Note

All claims expressed in this article are solely those of the authors and do not necessarily represent those of their affiliated organizations, or those of the publisher, the editors and the reviewers. Any product that may be evaluated in this article, or claim that may be made by its manufacturer, is not guaranteed or endorsed by the publisher.
